# Comparison of renal function after robot - assisted laparoscopic radical prostatectomy versus retropubic radical prostatectomy

**DOI:** 10.1590/S1677-5538.IBJU.2018.0103

**Published:** 2019

**Authors:** Giray Ergin, Omer Gokhan Doluoglu, Mustafa Kıraç, Muhammet Fatih Kilinc, Burak Köprü, Bugra Bilge Keseroglu, Mustafa Burak Hoscan

**Affiliations:** 1 Department of Urology Clinic, Yuksek Ihtisas University, Medical Faculty, Koru Hospital, Ankara, Turkey;; 2 Department of Urology Clinic, Ankara Training and Research Hospital, Ankara, Turkey;; 3 Department of Urology Clinic, Medstar Topcular Hospital, Antalya, Turkey

**Keywords:** Prostatic Neoplasms, Prostatectomy, Video-Assisted Surgery

## Abstract

**Purpose:**

To investigate the effect of robot assisted laparoscopic radical prostatectomy (RALP) and open retropubic radical prostatectomy (RRP) on early renal function in this study.

**Materials and Methods:**

Preoperative and postoperative urea, creatinine, Hb, eGFR values of patients who had undergone RALP and RRP with prostate cancer (PCa) diagnosis were recorded in our clinic. The percentages of change in these values are calculated. Preoperative and postoperative urea, creatinine, Hb and eGFR changes were compared with each other. Student-t test was used for intergroup comparison, and paired sample t test was used to compare changes between preoperative and postoperative values of the same group.

**Results:**

There were 160 and 93 patients in the RALP and RRP group, respectively. In the RALP group, postoperative urea and creatinine increased significantly compared to preoperative baseline values while eGFR was decreased (p = 0.0001, p = 0.001, p = 0.0001, respectively). Except for Hb in the RRP group, the changes in these values were statistically insignificant (p = 0.50, p = 0.75, p = 0.30, respectively).

**Conclusions:**

We should be more careful when we perform RALP in patients at risk of impaired renal function despite being a minimally invasive surgical method with superior visual characteristics.

## INTRODUCTION

Radical prostatectomy (RP) is a standard surgical treatment for clinically localized prostate cancer (1). Although robot-assisted laparoscopic radical prostatectomy (RALP) is increasingly common today, open retropubic radical prostatectomy (RRP) is still widely used. Because RALP does not exist in many centers. RALP is a minimally invasive method with high optical quality. RALP has fewer bleeding, fewer blood transfusions, less hospitalization, and excellent cosmetic results than RRP but oncological and functional outcomes are similar to RRP (2-5). Besides, the duration of the operation is longer in RALP. Longer operation time in RALP results in adverse physiological changes that are caused by the pneumoperitoneum and deep trandelenburg position. Renal blood flow and glomerular filtration rate (GFR) may be decreased by increased intraabdominal pressure (6-8).

Although there are many articles in the literature that compare the oncologic and functional outcomes of RRP and RALP, there is only one study comparing their effects on kidney function according to our knowledge. Interestingly, in this study, RRP has been shown to have more negative effects on renal function (9). We aimed to evaluate the effect of RRP and RALP on early renal function in our patient series in order to contribute to this gap in the literature.

## MATERIALS AND METHODS

We retrospectively reviewed the data of patients who had undergone RALP and RRP with prostate cancer diagnosis between January 2010 and June 2014 in our clinic after the local ethics committee approval (6.12.2017 / 299). All patients underwent RALP under general anesthesia. A Veress needle was inserted at the periumbilical position, pneumoperitoneum was established initially at 20 mmHg for adequate port positioning, and then lowered to 12-15 mmHg. Patients with end - stage renal insufficiency, and > ASA III score were excluded from the study.

Age, preoperative urea, creatinine, hemoglobin (Hb) and estimated glomerular filtration rate (eGFR) values of all patients were recorded. In addition, urea, creatinine, Hb, eGFR values of all patients at postoperative 12 hours were also recorded. Urea change percentages, creatinine change percentages, and eGFR change percentages were calculated in the postoperative period ([Last value - initial value] / initial value x 100). The time of the operations were also recorded. The operation time was defined as the time between first incision and the end of the operation.

eGFR was calculated using the 4-variable (age, sex, race, and serum creatinine) Modification of Diet in renal Disease Study equation: eGFR = 186 x serum creatinine x age x [0.742 if female] x [1.210 if African - American] (10).

Primary endpoint of this study was the comparison of the change percentage of eGFR. In addition, it was evaluated that whether acute kidney injury (AKI) developed in both groups according to KDIGO criteria. AKI was assessed as a postoperative increase in creatinine level of 0.3 mg/dL or greater within 48 hours (11).

The data analyses were performed with PASW 18 (SPSS / IBM, Chicago, IL, USA) software. Kolmogorov - Smirnov and P-P Plot tests were used to verify the normality of the distribution of continuous variables. The results were reported as mean ± SD, or in situations in which the distributions were skewed, as the median (minimum - maximum). Categorical variables were given as percentages. All statistical tests were two - tailed. Student-t test was used for the intergroup analysis of continuous variables. Categorical variables were analyzed with Chi square test. The difference between preoperative and postoperative values was assessed by paired samples t test. p < 0.05 was considered as statistically significant.

## RESULTS

Two hundred fifty three patients who met the study criteria were included in the study. 160 patients underwent RALP (Group-1) and 93 patients underwent RRP (Group-2). The mean age was 62.3 ± 6.4 in group 1 and 64.9 ± 5.4 in group 2 (p = 0.001).

Preoperative and postoperative age, urea, creatinine, hemoglobin, eGFR values, urea, creatinine, eGFR change rates, operative time and statistical comparisons of both groups are summarized in Table 1 and 2. When the two groups were compared, the percentages of eGFR changes were statistically significant (p = 0.009).

**Table 1:** Age, preoperative and postoperative data between the RALP and RRP patients.

	Group 1	Group 2	P value
Age	62.3 ± 6.4	64.9 ± 5.4	0.001*
Preop Urea (mg/dL)	16.1 ± 5.4	36.9 ± 11.4	0.0001*
Postop Urea (mg/dL)	13.5 ± 5.8	37.8 ± 11.8	0.0001*
Preop Creatinine (mg/dL)	0.94 ± 0.34	1.09 ± 0.20	0.0001*
Postop Creatinine (mg/dL)	1 ± 0.42	1.08 ± 0.22	0.52
Preop eGFR, mL/min/1.73 m²	93.1 ± 23	74.7 ± 15.1	0.0001*
Postop eGFR, mL/min/1.73 m²	88.6 ± 25.7	76.3 ± 19.7	0.0001*
Preop Hemoglobin (g/dL)	14.5 ± 1.2	14.6 ± 1.4	0.59
Postop Hemoglobin (g/dL)	12.1 ± 1.3	11.6 ± 1.5	0.008*
Operation time (minute)	166.9 ± 47.4	149.5 ± 21	0.0001*

* = Statistically significant

**Table 2:** The percentage changes of Urea, creatinine, eGFR before and after surgery.

	Group 1	Group 2	P value
∆ eGFR	-3.7 ± 19.8	3.07 ± 19.8	0.009*
∆ Urea	-13.5 ± 25.5	7.3 ± 39.4	0.0001*
∆ Creatinine	6.19 ± 19.7	0.32 ± 17.3	0.018*
∆ Hemoglobin	-16.5 ± 8.4	-20.6 ± 9.3	0.0001*

∆ = Percentage change

* = Statistically significant;

eGFR = Estimated glomerular filtration rate, (mL/min/1.73 m²)

Changes in preoperative and postoperative urea, creatinine, Hb and eGFR values in the RALP group were statistically significant. (p values: 0.0001, 0.001, 0.0001, 0.0001 respectively). In the RRP group, the changes in preoperative and postoperative values other than Hb were statistically insignificant. (p values: 0.5, 0.75, 0.0001 and 0.3 respectively) (Table-3).

**Table 3:** Intragroup comparison of eGFR, urea, creatinine levels and hemoglobin between preoperative and postoperative early periods (paired samples t-test).

	Group 1 (n=160)		p	Group 2 (n=93)		p
	Preoperative	Postoperative		Preoperative	Postoperative	
eGFR, (mL/min/1.73 m²)	93.1 ± 23	88.6 ± 25.7	0.0001*	74.7 ± 15.1	76.3 ± 19.7	0.30
Urea, (mg/dL)	16.1 ± 5.4	13.5 ± 5.8	0.0001*	36.9 ± 11.4	37.8 ± 11.8	0.50
Creatinine, (mg/dL)	0.94 ± 0.34	1 ± 0.42	0.001*	1.09 ± 0.20	1.08 ± 0.22	0.75
Hemoglobin, (g/dL)	14.5 ± 1.2	12.1 ± 1.3	0.0001*	14.6 ± 1.4	11.6 ± 1.5	0.0001*

* = Statistically significant

**Figure 1:**
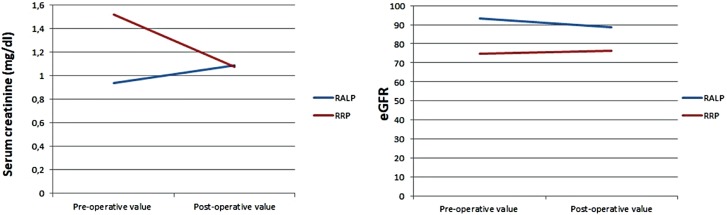
Changes in eGFR and creatinine in both groups.

**Figure 2:**
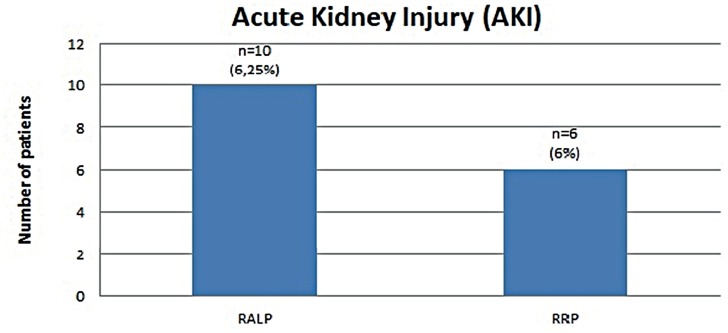
Incidence of patients with AKI in both groups.

While AKI was detected in 10 of 160 patients (6.4%) in Group-1, AKI was detected in 6 of 96 patients (6.5%) in Group-2 (p = 0.99). The changes in eGFR and creatine are shown in Figure-1, and the incidence of patients with AKI is seen in Figure-2.

## DISCUSSION

Robot-assisted laparoscopic radical prostatectomy has been increasingly performed as a minimally invasive surgical option with improved outcome compared with open or laparoscopic methods (12). Although RALP provides a superior point of view, pneumoperitoneum and trandelenburg position decrease renal blood flow and urine output by increasing intraabdominal pressure (6, 7, 13). There are many studies in the literature showing that the oncologic and functional outcomes of RALP and RRP are similar (2-5). However, to our knowledge, there is only one study comparing the effect of RALP and RRP on renal function. In this study, the authors compared a total of 307 patients (after propensity score matching) undergoing RALP and RRP and found that AKI was statistically higher in RRP group (5.5% vs. 10.4%, p = 0.044, respectively). The authors attributed this result to a higher blood loss during RRP and an increased risk of oxidative stress resulting from decreased renal blood flow. They also said that the blood transfusion given during RRP may be related with higher incidence of AKI (9). In this study, the authors did not compare early renal function but only the cases that developed AKI. In our study, AKI rates were similar in both groups.

In another study, significant changes were observed on postoperative 1, 3, and 7 days after robot - assisted laparoscopic radical prostatectomy and postoperative renal functions were found to be similar to baseline values at 30 days. The authors have said that RALP does not lead to long - term postoperative renal dysfunction. However, in this study only RLAP patients were included in the study, so no comparison was made with the RRP method (14).

In our study, we evaluated the effect of RALP and RRP on early renal function. Changes in eGFR, urea and creatinine levels in our study were significant in the RALP group but not in the RRP group. The changes in Hb levels were significantly decreased in both groups, while more blood loss was detected in the RRP group. When we looked at the percentages of change between the groups, the changes between the two groups were statistically significant. In the RALP group, in addition to the pneumoperitoneum, a more prominent trandelenburg position is required. These two conditions result in a more pronounced increase in central venous pressure and a more pronounced decrease in renal blood flow (6, 15-17).

It can be perceived as bias that in our study, preoperative age, urea, creatinine and eGFR values were different in both groups. However, although these values were better in the RALP group, they significantly changed when compared to the RRP group. This change is an indication of the effect of pneumoperitoneum and renal blood flow reduction on renal function during RALP.

Its retrospective nature and the lack of long - term follow-up of renal function are limititations of our study. However, it is one of the few studies evaluating the effect of RALP and RRP on early renal function.

## CONCLUSIONS

RALP is a minimally invasive surgical method that has been used increasingly widely, with superior visual characteristics. RRP is another proven surgical method with good functional and oncologic outcomes in experienced hands. We should be more careful when we are going to make RALP in patients with especially those at risk of impaired renal function. Prospective randomized studies with longer follow-up are needed to clarify this issue.

## ABBREVIATIONS

AKI: = Akut Kidney Injury
eGFR: = Estimated Glomerular filtration rate
Hb: = Hemoglobin
KDIGO: = Kidney Disease Improving Global Outcomes
RRP: = Open retropubic radical prostatectomy
PCa: = Prostate cancer
RALP: = Robot assisted radical prostatectomy
